# Superconducting nanowire single-photon detectors integrated with tantalum pentoxide waveguides

**DOI:** 10.1038/s41598-020-74426-w

**Published:** 2020-10-13

**Authors:** Martin A. Wolff, Simon Vogel, Lukas Splitthoff, Carsten Schuck

**Affiliations:** 1 Institute of Physics, University of Münster, Wilhelm-Klemm-Str. 10, 48149 Münster, Germany; 2 CeNTech – Center for Nanotechnology, Heisenbergstr. 11, 48149 Münster, Germany; 3 SoN – Center for Soft Nanoscience, Busso-Peus-Straße 10, 48149 Münster, Germany

**Keywords:** Nanophotonics and plasmonics, Superconducting devices, Quantum optics, Silicon photonics, Single photons and quantum effects, Photonic devices

## Abstract

Photonic integrated circuits hold great potential for realizing quantum technology. Efficient single-photon detectors are an essential constituent of any such quantum photonic implementation. In this regard waveguide-integrated superconducting nanowire single-photon detectors are an ideal match for achieving advanced photon counting capabilities in photonic integrated circuits. However, currently considered material systems do not readily satisfy the demands of next generation nanophotonic quantum technology platforms with integrated single-photon detectors, in terms of refractive-index contrast, band gap, optical nonlinearity, thermo-optic stability and fast single-photon counting with high signal-to-noise ratio. Here we show that such comprehensive functionality can be realized by integrating niobium titanium nitride superconducting nanowire single-photon detectors with tantalum pentoxide waveguides. We demonstrate state-of-the-art detector performance in this novel material system, including devices showing 75% on-chip detection efficiency at tens of dark counts per second, detector decay times below 1 ns and sub-30 ps timing accuracy for telecommunication wavelengths photons at 1550 nm. Notably, we realize saturation of the internal detection efficiency over a previously unattained bias current range for waveguide-integrated niobium titanium nitride superconducting nanowire single-photon detectors. Our work enables the full set of high-performance single-photon detection capabilities on the emerging tantalum pentoxide-on-insulator platform for future applications in integrated quantum photonics.

## Introduction

Quantum technology promises to overcome fundamental limitations of classical information processing, communication and sensing^1^. Single-photons are a particularly favorable choice as information carriers in quantum technology because they are fast, easy to manipulate and show negligible decoherence^2^. Single-photon-based quantum technologies significantly benefit from photonic integrated circuit (PIC) implementations, which leverage modern nanofabrication methods for the realization of complex quantum optical networks on a stable and scalable platform^3^. One of the key requirements for realizing the promises of photonic quantum circuits, however, is the integration of PICs with efficient single-photon detectors in order to provide nonlinear optical functionality^4^ and efficient measurement capabilities^5^. Waveguide-integrated superconducting nanowire single-photon detectors (SNSPDs) are a rapidly developing technology to satisfy these measurement-demands of nanophotonic quantum circuits^6,7^. SNSPDs offer efficient photon counting over an extremely broad wavelength range and show outstanding performance in terms of speed, timing resolution and dark count rates^8^. By integrating SNSPDs with optical waveguides, an efficient interface between photonic quantum circuits and detectors is straightforwardly achieved, thus overcoming scaling issues with off-chip detector solutions that are currently being used for the overwhelming part of quantum optics experiments^9–13^.

An integrated quantum photonic platform with waveguide-coupled SNSPDs faces two crucial challenges for future quantum technology applications: On the one hand, the detectors need to enable single-photon counting with high signal-to-noise ratio as well as high operating speeds. On the other hand, the nanophotonic platform has to provide low-loss transmission, a wide band gap, high refractive index contrast and compatibility with advanced nanofabrication processes, while ideally also featuring parametric frequency conversion capabilities via strong optical nonlinearities and high thermo-optic stability. Several combinations of superconducting and dielectric materials are currently being considered^6,7,14–19^ to simultaneously satisfy the detector and PIC demands, however none is emerging as a favorite with respect to the above requirements.

Here we show that integrating SNSPDs made from niobium titanium nitride (NbTiN) thin films with tantalum pentoxide (Ta_2_O_5_) photonic integrated circuits is extremely well-suited to satisfy both detector and PIC demands of future photonic quantum technology implementations. NbTiN SNSPDs have shown outstanding performance in terms of both high-speed detection, due to the low kinetic inductance of NbTiN nanowires^20,21^, as well as high signal-to-noise ratio^22^ as a result of extremely low dark count rates^23,24^, suitable even for most demanding applications^25–27^. Additionally, NbTiN detectors have recently shown high detection efficiencies and low timing jitter simultaneously, allowing for extremely precise photon-correlation measurements^28,29^. The polycrystalline structure of NbTiN enables high yield production of state-of-the-art performance SNSPDs in established nanofabrication processes^30,31^ on various photonic platforms^16,27^.

Tantalum pentoxide is well-known for its excellent optical properties^32–34^ and finds increasing recognition as a promising high-refractive index dielectric for realizing photonic integrated circuits^35,36^. The compatibility of Ta_2_O_5_ thin-films with modern nanofabrication routines is evident from its established use in complementary metal oxide semiconductor processes, exploiting the material’s high relative permittivity^37^, which will also benefit on-chip electric read-out circuitry for SNSPDs, e.g. by reducing the length of impedance matching tapers^38^. Ta_2_O_5_ further features a wide band gap and lower density of impurities^39^, resulting in significantly lower optical loss at visible and near-infrared wavelengths as compared to other nanophotonic platforms such as silicon nitride (Si_3_N_4_). Notably, Ta_2_O_5_ also offers very attractive material properties for realizing single-photon sources. One of the material’s distinguishing properties is an extremely low level of autofluorescence^40,41^, which strongly benefits the integration of optically excited solid-state quantum emitters. The third-order optical nonlinearity of Ta_2_O_5_ (up to 1.4 × 10^−18^ m^2^/W^42^) is significantly larger than those of Si_3_N_4_ (2.4 × 10^−19^ m^2^/W^43^) and SiO_2_ (2.8 × 10^−20^ m^2^/W^44^) which allows for implementing efficient four wave mixing processes^36,42,45,46^. Nonlinear optics applications in the Ta_2_O_5_ material system will further benefit from deposition of relatively thick films with minimal internal stress, thus allowing for greater design freedom in engineering anomalous group velocity dispersion as desired e.g. for frequency comb generation. Importantly in this regard is also the record low thermo-optic coefficient (5.8 × 10^−6^/K^47^), which is smallest among all current nanophotonic material systems (e.g. Si_3_N_4_ 2.5 × 10^−5^/K^48^, SiO_2_ 1.0 × 10^−5^/K^48^), benefitting cryogenic applications and allowing for stable operation of high-Q resonators even at high optical power levels^49^. However, to leverage these attractive properties of Ta_2_O_5_ for photonic quantum technology, an integrated single-photon detection solution is needed.

We demonstrate the integration of NbTiN SNSPDs with Ta_2_O_5_ nanophotonic circuits in a travelling wave geometry^6,14^, which enables efficient absorption of photons guided in PICs via a low-loss interface without the need for resonant nanostructures. For this purpose, we developed a room-temperature magnetron sputtering process for depositing NbTiN thin films onto Ta_2_O_5_-on-insulator substrates produced via DC physical vapor deposition. The high refractive index contrast of Ta_2_O_5_-on-insulator allows us to fabricate nanophotonic devices with a small footprint and with minimal optical loss on a single monolithic silicon chip. The use of thin NbTiN nanowires further enables the realization of SNSPDs with extremely low kinetic inductance, thus facilitating high detector operating speeds via sub-ns decay times. Importantly, our Ta_2_O_5_ waveguide-integrated SNSPDs show excellent performance in terms of signal-to-noise ratio, i.e. detected photon count rate versus dark count rate, as evident from saturated internal quantum efficiency over a large bias current range. Such signal-to-noise ratio had remained elusive for telecom wavelengths photons in the NbTiN material system thus far. Consequently, we can operate the detectors in a regime where dark count rates are as low as a few counts per second (cps) and fluctuations in the bias current only have a negligible effect on the saturated detection efficiency, which remains maximal at $\left( {75 \pm 9} \right)$% for a 70 µm long SNSPD. We further show that changing the length of our detectors leads to an increase of the detection efficiency without compromising the saturation behavior and therewith the overall signal-to-noise ratio behavior. Lastly, we note that our NbTiN-SNSPDs on Ta_2_O_5_ waveguides show attractive timing performance with electrical readout-limited jitter values as low as 28 ps using room-temperature signal amplification. Achieving these high speed and high signal-to-noise ratio detector performance on a PIC platform, which offers superior prospects for integrated quantum photonics, will enable a multitude of applications with exciting long-term perspectives for increasingly complex quantum technology implementations.

## Results and discussion

### Integration of NbTiN SNSPDs with Ta_2_O_5_ PICs

**Figure 1:**
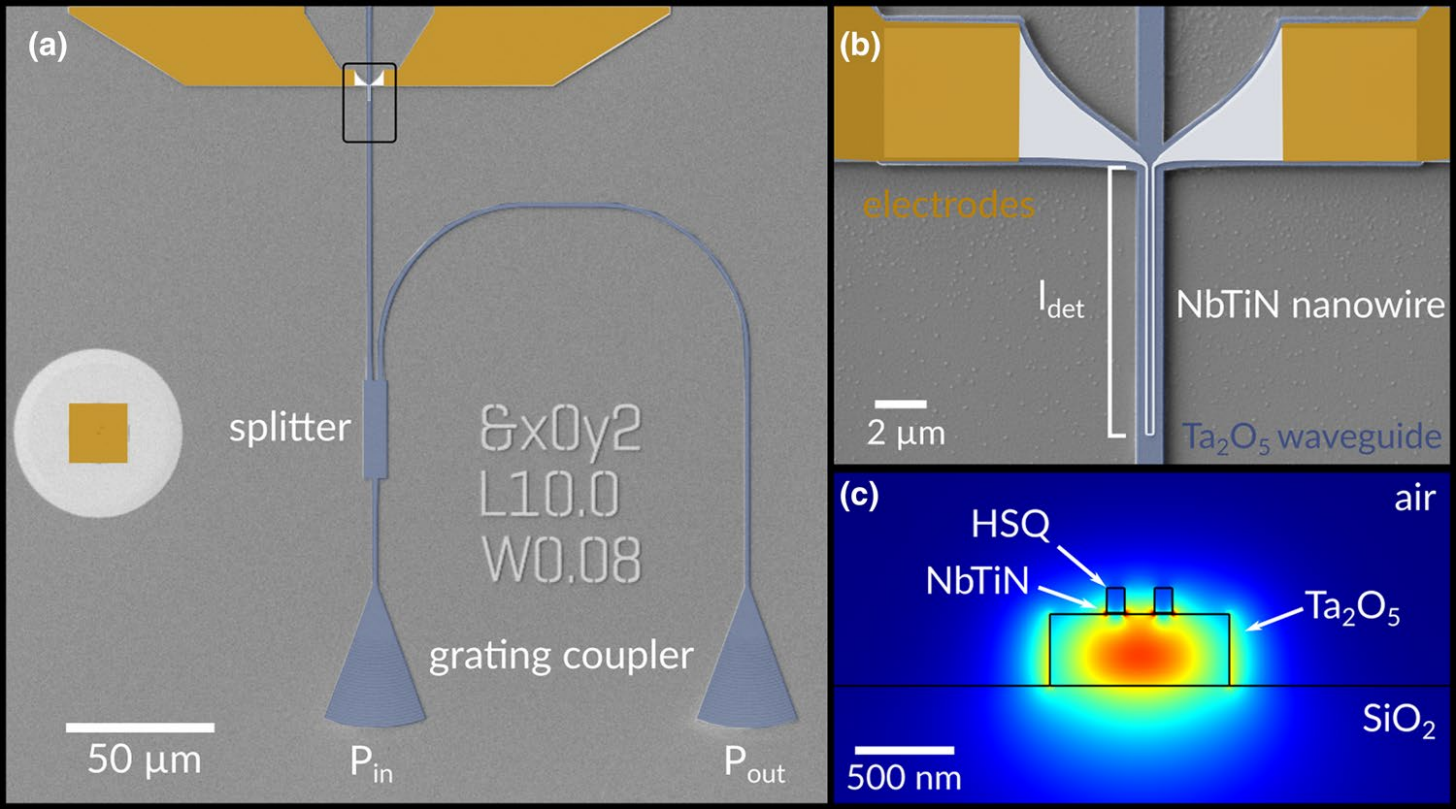
(**a**) Nanophotonic circuit for characterization of the SNSPD. Two grating couplers are used for the alignment and calibration of the chip inside the cryostat, with input power port P_in_ and output power port P_out_. A multi-mode interference device serves as an optical power splitter of the incoming light. (**b**) Scanning electron micrograph (false color) of a fabricated Ta_2_O_5_ SNSPD device. The length of the nanowire, $l_{\text{nw}}$, is equal to approximately twice the detector length, $l_{\det}$. (**c**) Illustration of a supported TE_00_ mode inside the tantalum pentoxide waveguide on an insulating silicon dioxide on silicon substrate with the niobium titanium nitride nanowire and hydrogen silsesquioxane resist on-top (simulated using the finite element method). The gap between the NbTiN nanowires is 150 nm.

We fabricate waveguide-integrated SNSPDs by sputtering a thin film of NbTiN from an alloy target at room temperature in an ultra-high vacuum environment onto 360 nm thick Ta_2_O_5_ films on thermally grown silicon dioxide (SiO_2_) on silicon (Si) wafers. Both superconducting nanowires and nanophotonic devices are fabricated in a top-down multi-layer process with high overlay accuracy using electron beam lithography and reactive ion etching (see Methods for further details). Electrical access to the superconducting nanowires is established via electrode pads and photonic access to the PIC via optical grating couplers, as shown in Fig. 1. The grating couplers are optimized for operating wavelengths in the telecommunication C-band through adjusting the grating period and fill factor. Photons propagate in the fundamental TE_00_ mode through low-loss single-mode waveguides (Fig. 1c) to the detector region, where they are absorbed along their direction of propagation in the U-shaped NbTiN nanowire, preferably at the edges of the nanowire, located in the evanescent field on top of the Ta_2_O_5_-waveguide. We use multi-mode interference (MMI) devices to guide a well-controlled fraction of the photon flux propagating towards the detector in order to calibrate the number of photons arriving at the NbTiN nanowire. We further taper our photonic waveguides down from a width of 1.2 to 0.9 µm to enhance the absorption at the detector. The chip is mounted in a cryogenic measurement setup, which allows us to engage the on-chip electrodes and optical grating couplers with a radiofrequency (rf) probe and an optical fiber array, respectively. We use the fiber array to both send light from swept continuous wave (cw) and pulsed laser sources to the chip as well as for collecting light from the reference output of the MMI device. The photon flux at the Ta_2_O_5_-waveguide-integrated SNSPD can then be estimated from the optical power measured at the reference output^50^

1 $$\phi = \frac{\lambda }{h \cdot c}\sqrt {0.5 \cdot P_{in} \cdot P_{out} }$$

where $\lambda$ denotes the input laser wavelength, $h$ is Planck’s constant, $c$ is the speed of light and $P_{in}$ and $P_{out}$ are the powers measured at the input and output grating couplers, respectively. Hence, measuring input and output power levels with a calibrated power meter allows us to precisely adjust the photon flux in the detector region using calibrated optical attenuators.

We operate the devices at temperatures of 1.3 K inside a closed-cycle. He-4 cryostat and bias the superconducting NbTiN nanowires with a low-noise current source via a bias-T and the rf-probe. We record the electrical response of the nanowires to an optical input with a frequency counter or on an oscilloscope, which allows us to determine the detector count rate and timing performance, respectively.

### Signal-to-noise ratio

High detection efficiency is a sought-after characteristic of any single-photon counting solution. However, most applications can only take full advantage of measuring the count rate, i.e. the signal, of incident photons with high detection efficiency, when a low rate of dark counts, i.e. noise, is achieved simultaneously. NbTiN was shown to benefit such high signal-to-noise ratio operation^22,51^ because typical detection efficiencies are only marginally smaller than for the most efficient SNSPDs^28,52^, while dark count rates can be orders of magnitude smaller than for high-efficiency SNSPDs made from other superconducting material systems^23^. Here we show how low dark count rate and high detection efficiency for photons in Ta_2_O_5_-waveguides are achieved simultaneously by realizing SNSPDs that show saturated internal detection efficiency over a wide bias current range.

**Figure 2:**
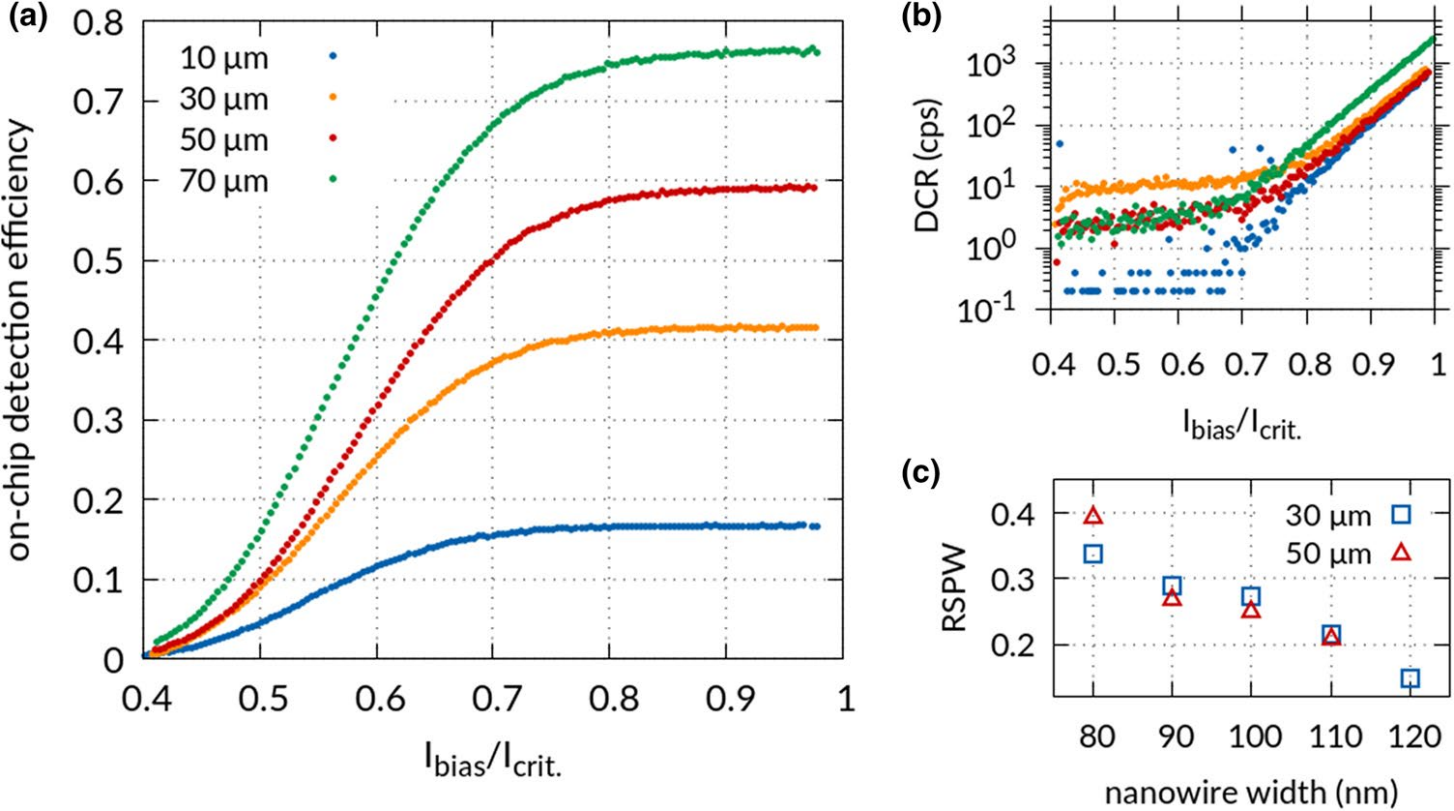
(**a**) On-chip detection efficiency of the Ta_2_O_5_ waveguide-integrated NbTiN SNSPD with 90 nm wide nanowires of 10 µm (blue), 30 µm (yellow), 50 µm (red) and 70 µm (green) detector length and (**b**) their corresponding dark count rate versus the normalized bias current relative to the respective experimental critical current. (**c**) Relative saturation plateau width of Ta_2_O_5_ waveguide-integrated NbTiN SNSPDs using the definition in Eq. (2) (see main text) for 30 µm (blue), 50 µm (red) detector length.

In order to find optimally performing devices we characterize a large number of detectors on a chip containing more than 200 waveguide-integrated SNSPDs with systematically varied geometries, i.e. nanowire widths, lengths, and gaps. We determine the on-chip detection efficiency, $OCDE$, for each device by measuring the count rate, $CR$, as a function of bias current for a calibrated input photon flux, $\phi$, from an attenuated 1550 nm wavelength cw-laser source. Similarly, we measure the dark count rate, $DCR$, of our detectors when switching off the laser source but maintaining typical working conditions otherwise, i.e. straylight from the laboratory environment may be efficiently guided towards the SNSPD^53^. We thus obtain the $OCDE = \left( {CR - DCR} \right){/}\phi$ as shown in Fig. 2a for 90 nm wide nanowire detectors of different lengths. Importantly, we find long plateaus of constant $OCDE$ with increasing bias current for all nanowire lengths, indicating saturation of the internal quantum efficiency even at bias currents far below the critical current for our measurements. This behavior enables operation with high signal-to-noise ratio because it allows for biasing the detector far below the critical current without a decrease in the $OCDE$. Operating at such lower bias currents prevents excessive dark count rates, which reduces to a few counts per second for our devices shown in Fig. 2b, thus benefitting the signal-to-noise ratio. We further find that the relative width of the OCDE saturation plateau correlates with nanowire width, as shown in Fig. 2c. Here we define the relative saturation plateau width^30^

2 $${\text{RSPW}} = \left( {{\text{I}}_{{\text{c}}} - {\text{I}}_{{{\text{sat}}}} } \right)/{\text{I}}_{{\text{c}}}$$

where ${\text{I}}_{{\text{c}}}$ is the experimentally determined critical current in the absence of light of the nanowire and ${\text{I}}_{{{\text{sat}}}}$ denotes the bias current at 90% of the saturated $OCDE$. As the nanowire width decreases from 120 to 80 nm we observe an increase in relative saturation plateau width up to 39% of the bias current range for 50 µm long detectors. We interpret this as the result of suppressing the superconductivity in larger fractions of the nanowire as the width reduces. On the other hand, the relative saturation plateau width shows no significant dependence on nanowire length, as shown in Fig. 2c for 30 µm and 50 µm long detectors, which indicates that imperfections originating from the fabrication process only have a minor influence.

In order to determine a detector design with optimal signal-to-noise ratio on Ta_2_O_5_-waveguides we further vary the detector length, because longer nanowires should lead to larger absorption probabilities for single-photons in the case of the traveling wave geometry employed here^14,54^. As shown in Fig. 2a for SNSPDs made from 90 nm wide nanowires we find an $OCDE = \left( {75 \pm 9} \right) \%$ at a bias current of 85% of ${\text{I}}_{{\text{c}}}$ for a detector length of 70 µm. The benefit in signal-to-noise ratio is highlighted by a reduction in dark count rate over two orders of magnitude as one varies the bias current over the saturation plateau width from 95 to 75% of the critical current without compromising the detection efficiency. These results further illustrate that waveguide-integrated NbTiN SNSPDs on Ta_2_O_5_-waveguides allow for extremely stable operating conditions over the saturation plateau regime where fluctuations in the bias current have negligible effects on the detection efficiency, thus reducing sensitivity to external perturbations.

### Dynamic detector response

**Figure 3:**
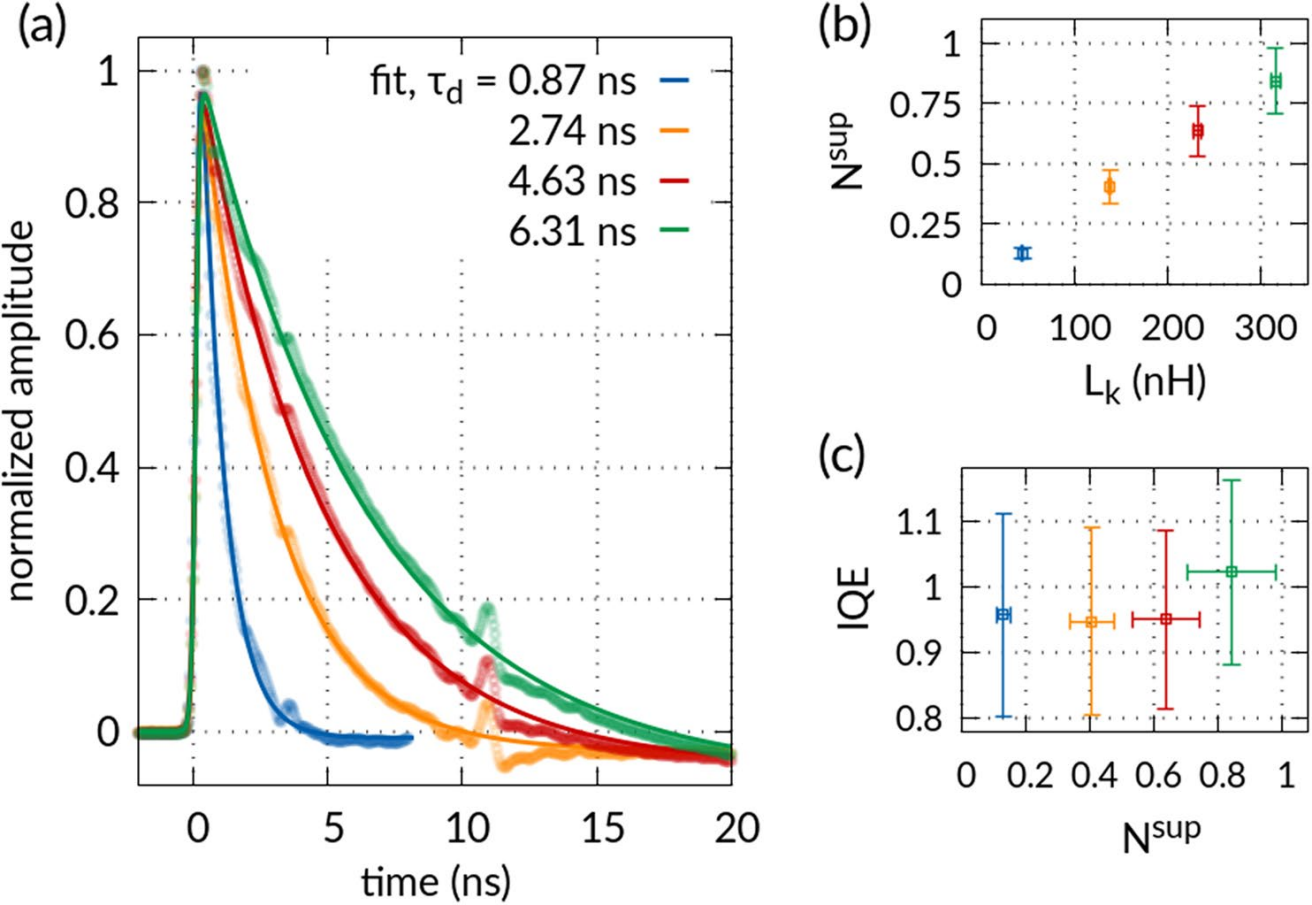
(**a**) Average of 10 k oscilloscope traces of the electrical response of waveguide-integrated SNSPDs for 90 nm wide nanowires of 10 µm (blue), 30 µm (yellow), 50 µm (red) and 70 µm (green) detector length. Fits to the data are calculated using Eq. (3) (see main text). (**b**) The suppressed number of squares, which are switching to the normal conducting state, versus kinetic inductance for SNSPDs with different lengths. (**c**) The internal quantum efficiency (IQE), as calculated by dividing the OCDE (see Fig. 2a) by the absorption efficiency (see main text), versus the calculated suppressed number of squares.

Timing accuracy and photon counting rate are other single-photon detector performance parameters of major importance for future quantum technology, e.g. for fast quantum communication^55^ and active feed-forward schemes^56^. Here we demonstrate that the dynamical response of NbTiN SNSPDs on Ta_2_O_5_-waveguides shows very appealing performance for future integrated quantum photonics. We characterize the dynamic response of our detectors by recording the electrical output signals upon photon detection on an oscilloscope. Firstly, we assess the detector speed by forcing the nanowire into a transient resistive state upon absorbing a photon and determine the rise and decay times of the electrical output signal. Representative voltage signals that are normalized and averaged over 10 k individual detection events are shown in Fig. 3a for detectors of length $l_{{\det}}$ over several tens of micrometer. Note that the length of a nanowire $l_{{{\text{nw}}}}$ is equal to approximately twice the detector length $l_{{\det}}$, due to the U-shaped geometry (see Fig. 1b). We fit the experimental data using the functional behavior^57^

3 $${\text{f}}\left( {\text{t}} \right) \propto \frac{{\exp \left( { - \frac{{\text{t}}}{{\uptau _{{\text{d}}} }}} \right)}}{{1 + \exp \left( { - 2\ln \left( 9 \right)\frac{{\text{t}}}{{\uptau _{{\text{r}}} }}} \right)}}$$

where $\uptau _{{\text{d}}}$ is the decay time, i.e. the time interval over which the signal decreases to 1/e of its maximum value, and $\uptau _{{\text{r}}}$ the rise time of the detector, i.e. the time interval in which the rising edge of the signal increases from 10 to 90% of the maximum signal amplitude. We find that the decay time of our 90 nm wide nanowires decreases with the length of the detector, reaching $\left( {869 \pm 11} \right) {\text{ps}}$ for a 10 µm long detector. The increase of the decay time with detector length is due to an increase of the nanowire’s kinetic inductance $L_{{\text{k}}} =\uptau _{{\text{d}}} \cdot {\text{R}}_{{\text{L}}}$, where ${\text{R}}_{{\text{L}}}$ denotes the load impedance. The normal state resistance of the nanowire region in which superconductivity is suppressed after a photon was absorbed then follows from the ratio of decay and rise times of the output voltage pulse according to $R_{{\sup}} = L_{{\text{k}}} {/}\uptau _{{\text{r}}}$, where $\uptau _{{\text{r}}}$ is in the range of $\left( {314 - 340} \right) {\text{ps}} \pm 52 \;{\text{ps}}$ for our 10 µm to 70 µm long NbTiN SNSPDs. Through comparison with the resistivity of the entire detector in the normal state, $R_{{{\text{tot}}}}$, i.e. as extracted from the I-V-characteristics at the operating temperature, we can estimate in what fraction of the nanowire the superconductivity is suppressed during a photon detection event. Here, it is convenient to divide the nanowire into squares^58^ of lateral length equal to the nanowire width, $w$, such that the total number of squares $N^{{{\text{tot}}}} = l_{{{\text{nw}}}} {/}w$. Upon photon detection superconductivity will only be suppressed in a fraction $N^{\sup }$ of these squares yielding the corresponding normal state resistance $R_{{\sup}}$ ^57^. For our NbTiN SNSPDs we find that less than one square, $N^{\sup } = N^{{{\text{tot}}}} \cdot \frac{{R_{{\sup}} }}{{R_{{{\text{tot}}}} }}$, switches to the normal state when absorbing a telecom wavelength photon, which we show in Fig. 3b in dependence of the kinetic inductance for 10 µm to 70 µm long detectors. We observe that the effective number of squares in the normal state for a switching event increases with the kinetic inductance of the detector, which indicates that the effective size of the hot-spot^59,60^ that is created upon photon absorption increases with the kinetic inductance of the nanowire. However, this dependence does not alter the bias current range and therewith the RSPW over which saturated detection efficiency is observed (Fig. 2), which is independent of the fraction $N^{\sup }$ and thus the effective hot-spot area. An optical transmission measurement of the absorption coefficient of our NbTiN nanowires yielded an attenuation of $\left( {0.0826 \pm 0.0092} \right)\;{\text{dB/}}\upmu {\text{m}}$ for light at telecom wavelengths. We thus extract the internal quantum efficiency (IQE) from the saturated OCDE measurement (Fig. 2a) and find that the internal quantum efficiency of our detectors is consistent with unity (see Fig. 3c).

**Figure 4:**
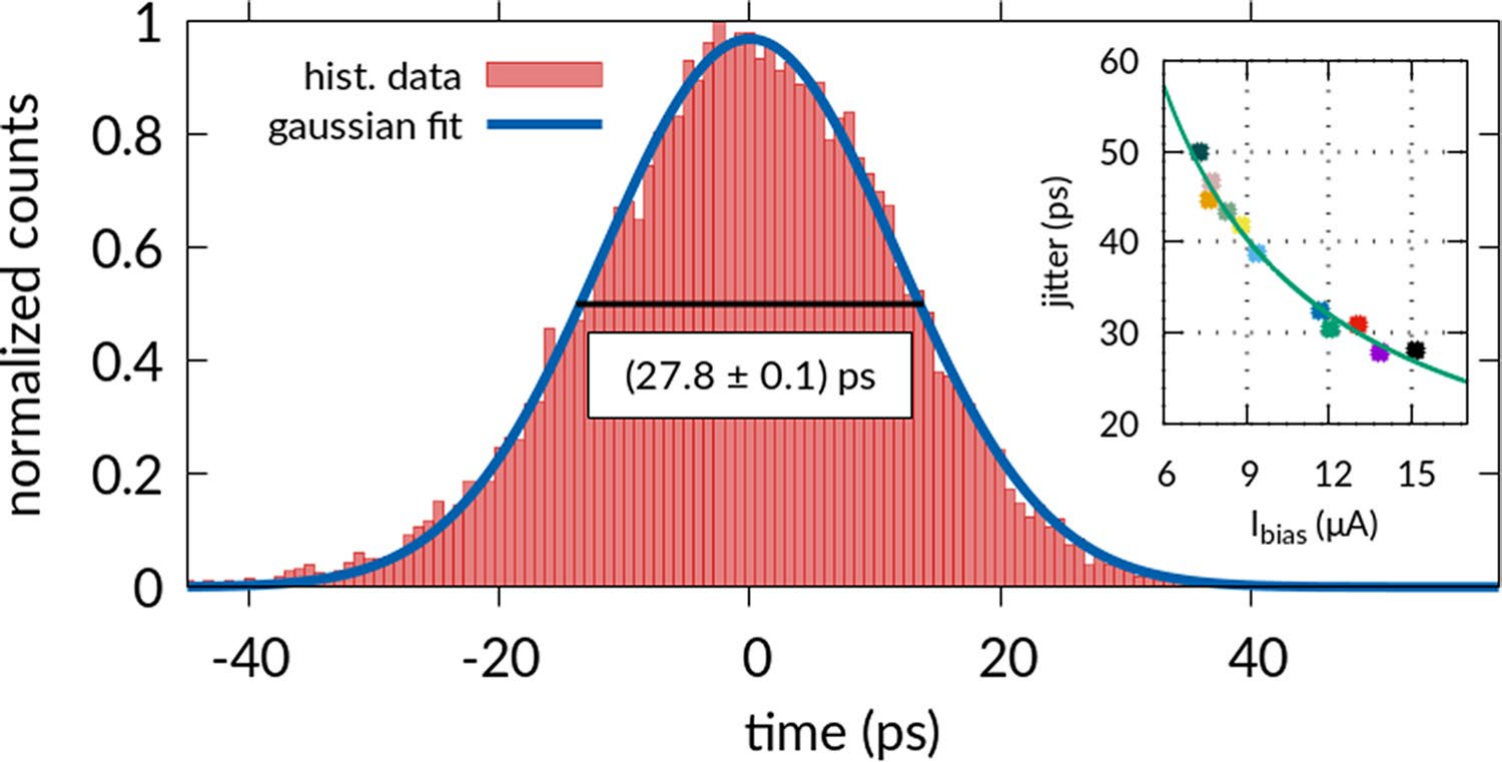
Histogram of temporal variations in the latency between the arrival of a photon at the nanowire and a corresponding electrical detector output pulse (jitter measurement) for an SNSPD with a detector length of 30 µm and a 110 nm wide nanowire. A fit to the data with a Gaussian function reveals a FWHM of $\left( {27.8 \pm 0.1} \right) {\text{ps}}$. Inset: Dependence of the FWHM jitter value on bias current for several SNSPDs with varying geometry, resulting in different detector critical currents. Each device was biased at 90% of the experimental critical current. A fit to the data reveals a $1/I_{{{\text{bias}}}}$-dependence of the jitter, indicating a large contribution from the electrical readout circuit.

Another important aspect of the dynamic response of SNSPDs is their timing accuracy, i.e. variations in the latency time between absorbing an incoming photon and producing an electrical output pulse. We investigate this detector jitter for our novel material combination of NbTiN nanowires on Ta_2_O_5_-waveguides by performing start-stop measurements with a 1550 nm wavelength pulsed laser source operating at 40.125 MHz repetition rate in the following manner. We use calibrated optical attenuators to set the average number of photons per pulse that arrive at the nanowire to $0.002\;{\text{photons/pulse}}$. We operate the detector at a bias current of 90% of the critical current and set the trigger level for electrical output pulses to 50% of the maximum signal height. We use this electrical SNSPD output signal as start trigger and the electrical reference output of the laser as stop signal. We then record the time interval between start and stop signals, which is shown as a histogram in Fig. 4. The jitter is then determined using a gaussian function, resulting in a full-width at half maximum of $\left( {27.8 \pm 0.1} \right) {\text{ps}}$ for a 110 nm wide nanowire with a detector length of 30 µm, where we extract the error from a nonlinear least-square fit. We note that this jitter value constitutes the convolution of the SNSPD-jitter and the inherent jitter of the signal derived from the laser head. We determine the latter in an independent self-referencing measurement between far-separated laser pulse signals as 1.5 ps, which is small relative to the SNSPD-jitter. Notably, our jitter value for SNSPDs on Ta_2_O_5_-waveguides is small compared to previously reported values for NbTiN-SNSPDs using signal amplification at room-temperature^21,23,25,61,62^. However, using cryogenic low noise amplifiers we believe that this value can further be reduced, as previously demonstrated for NbTiN-SNSPDs yielding sub 10-ps jitter^29^.

We further determine jitter values for a larger number of detectors that differ in geometry, with nanowire widths ranging from 80 to 120 nm and detector lengths of 10 µm to 100 µm (Fig. 4 inset), each of which we biased at 90% of their respective experimental critical current. We find that the overall jitter data shows a $1/I_{{{\text{bias}}}}$ dependence across all tested nanowire detector devices^63^. This implies that thermal noise from room-temperature electronic components accounts for a significant contribution to the overall jitter. We anticipate that this setup specific performance can be reduced by employing cryogenic amplification^28,30^ rather than low noise room-temperature amplifiers as it is currently the case for our measurement system. Further improvements in timing performance may be achievable when producing thicker superconducting films or even wider nanowires that allow for operating the SNSPDs at larger bias currents.

## Conclusion

Our results show that the integration of NbTiN nanowires with Ta_2_O_5_ nanophotonic circuitry meets the demands of next generation platforms for nanophotonic quantum technology with integrated SNSPDs that realize high on-chip detection efficiencies and excellent timing performance at telecommunication wavelengths.

In our experiments we find Ta_2_O_5_-waveguide-integrated SNSPDs that yield efficiencies of up to 75%, decay times below 1 ns and timing jitter as low as 28 ps. Wide plateaus of bias-current saturated internal detection efficiency at 1550 nm wavelength, which had not yet been demonstrated with waveguide-integrated NbTiN SNSPDs, enable us to operate the detectors in a high signal-to-noise ratio regime, where low dark count rate and high detection efficiency are achieved simultaneously.

We anticipate that the wide-band transparency of Ta_2_O_5_^35^ can be exploited for implementing integrated quantum photonics experiments in the visible wavelength range with further improvements in detection efficiency, such as longer plateaus of saturated internal OCDE, because the sensitivity of SNSPDs increases for shorter wavelengths^64^. The visible wavelength range is of particular relevance for future integrated quantum photonic applications exploiting single-photon generation from waveguide-coupled quantum emitters^65–67^, which will tremendously benefit from the low intrinsic autofluorescence^40,41^ and absorption^39^ of the tantalum pentoxide material system. Other attractive possibilities for quantum light generation in nanophotonic Ta_2_O_5_ devices arise from the material’s strong optical nonlinearity and small thermo-optic coefficient, which can be exploited for spontaneous four wave mixing^42,45,46^ alongside integrated SNSPDs. In combination with passive and active linear optical circuit components, such as high-quality factor micro-ring resonators and optomechanical phase shifters^34^, respectively, our NbTiN SNSPDs provide comprehensive functionality, essential for integrated quantum photonic technology that allows for scaling to large system size through straightforward device replication in modern silicon-based nanofabrication processes.

## Methods

### Device fabrication

We fabricate devices in a multi-layer process with three exposures in a 100 kV electron beam lithography (EBL) system (Raith EBPG5150). The first EBL-step consists of patterning alignment markers and contact pads in positive-tone poly methyl methacrylate (PMMA, solid content 4.5%) resist. After development in methyl isobutyl ketone and isopropyl alcohol we deposit a 5 nm Cr adhesion layer and 100 nm gold (Au) in electron-beam evaporation followed by lift off in acetone. In the second EBL-step we pattern the detector nanowires and their leads to the contact pads using negative-tone hydrogen silsesquioxane resist and apply electron beam dose corrections to avoid proximity effects. After development in tetramethylammonium hydroxide-based developer the pattern is transferred to the NbTiN layer in a timed reactive-ion etching step employing sulfur hexafluoride (SF6) chemistry in an Ar atmosphere. In the third EBL-step we employ the negative-tone photoresist ma-N 2403 for patterning photonic circuits. We achieve high overlay accuracy by aligning the patterns for each photonic circuit device to the same markers used in the previous step for defining the respective nanowire devices (see Fig. 1b). Following development in tetramethylammonium hydroxide-based developer the waveguide patterns are transferred to the Ta_2_O_5_ film via timed fluorine-based reactive-ion etching in an Ar atmosphere. The process concludes with a resist stripping procedure in oxygen plasma.

### Electrical and optical setup

All devices were characterized in a closed-cycle He-4 cryostat reaching a base temperature of 1.3 K. We efficiently thermalize our devices by introducing helium into the sample chamber in order to promote heat exchange. We produce stable bias currents for our SNSPDs from a voltage source (Keithley 2450) connected to a $1\,{\text{M}}\Omega$ resistor in series and followed by two low pass filters that attach to the DC port of a bias tee, which is connected to the sample via a coaxial cable and the radiofrequency probe inside the cryostat. The voltage response of the nanowire is read out via the RF port of the bias tee. We use two low noise amplifiers (ZFL-1000 LN, Mini-Circuits) connected in series for signal amplification before recording the dynamic response on an oscilloscope and extracting signal-time-traces and histograms of variations in signal latency (jitter). We characterized the − 3 dB cut-off of the amplifier chain to 1.82 GHz and account for possible rise time variations due to amplifier filtering in the error of t_rise_. For photon counting applications, we use a universal frequency counter and use calibrated attenuators to set a photon flux of 100 k photons per second, which is far below the inverse of the reset time of the detector and only has a negligible effect on the latching behavior of the nanowires (< 3% offset in the ${\text{I}}_{{\text{b}}} /{\text{I}}_{{\text{c}}}$ normalization in Fig. 2a). Here we take insertion loss at each optical component into account, which have been calibrated independently, but assume that input and output grating couplers have similar performance (optical reciprocity) and waveguide losses are negligible.

### Ultra-thin NbTiN film growth

For the deposition of the superconducting film we employed a DC magnetron sputtering process using a Nb_0.7_Ti_0.3_ alloy target in an Ar/N_2_ atmosphere of $3\;{\text{mTorr}}$ and apply a power of 300 W to the target. We maintain the process chamber at a base pressure below $5{ } \times 10^{ - 9} \;{\text{Torr}}$ to guarantee ultra-clean deposition conditions. We further employ a 50 W substrate bias to improve NbTiN thin-film growth at room temperature^68^. The absolute thickness of our NbTiN film is $\left( {6.4 \pm 0.3} \right)\;{\text{nm}}$, as determined from the sputter deposition rate.
